# Whole Genome Analysis of Epidemiologically Closely Related *Staphylococcus aureus* Isolates

**DOI:** 10.1371/journal.pone.0078340

**Published:** 2013-10-21

**Authors:** Maarten Schijffelen, Sergey R. Konstantinov, Gérard Lina, Iris Spiliopoulou, Engeline van Duijkeren, Ellen C. Brouwer, Ad C. Fluit

**Affiliations:** 1 Department of Medical Microbiology, University Medical Center Utrecht, Utrecht, The Netherlands; 2 Laboratoire de Bactériologie - CNR des Staphylocoques, University Claude Bernard, Lyon 1, Lyon, France; 3 Department of Microbiology, School of Medicine University of Patras, Patras, Greece; 4 Department of Infectious Diseases and Immunology, Faculty of Veterinary Medicine, University Utrecht, The Netherlands; 5 Centre for Infectious Disease Control Netherlands, Institute of Public Health and Environment, Bilthoven, The Netherlands; University of Liverpool, United Kingdom

## Abstract

The change of the bacteria from colonizers to pathogens is accompanied by a drastic change in expression profiles. These changes may be due to environmental signals or to mutational changes. We therefore compared the whole genome sequences of four sets of *S. aureus* isolates. Three sets were from the same patients. The isolates of each pair (S1800/S1805, S2396/S2395, S2398/S2397, an isolate from colonization and an isolate from infection, respectively) were obtained within <30 days of each other and the isolate from infection caused skin infections. The isolates were then compared for differences in gene content and SNPs. In addition, a set of isolates from a colonized pig and a farmer from the same farm at the same time (S0462 and S0460) were analyzed. The isolates pair S1800/S1805 showed a difference in a prophage, but these are easily lost or acquired. However, S1805 contained an integrative conjugative element not present in S1800. In addition, 92 SNPs were present in a variety of genes and the isolates S1800 and S1805 were not considered a pair. Between S2395/S2396 two SNPs were present: one was in an intergenic region and one was a synonymous mutation in a putative membrane protein. Between S2397/S2398 only one synonymous mutation in a putative lipoprotein was found. The two farm isolates were very similar and showed 12 SNPs in genes that belong to a number of different functional categories. However, we cannot pinpoint any gene that explains the change from carrier status to infection. The data indicate that differences between the isolate from infection and the colonizing isolate for S2395/S2396 and S2397/S2398 exist as well as between isolates from different hosts, but S1800/S1805 are not clonal.

## Introduction

*Staphylococcus aureus* generally colonizes human skin and mucous membranes. The anterior nares are most frequently the site of carriage [1,2]. Approximately 30% of the general population will be colonized by *S. aureus* at one time point [3] and three types of carriership can be distinguished: persistent carriers (~20%), intermittent carriers (~30%) and non-carriers (~50%) [3-6]. 

Nasal colonization by *S. aureus* is one of the most important factors in the pathogenesis of nosocomial infections, mainly in surgical site and vascular device related infections [7-10]. Staphylococcal infections normally occur when the mucosal or skin barrier breaches, e.g., by scratching, mechanical stress or surgery, thereby allowing access to the adjoining tissues or the bloodstream [11].

The change of the bacteria from colonizers to pathogens is accompanied by a drastic change in expression profiles [12]. Similar changes can be expected when bacteria change hosts. These changes in expression profile may be due to environmental signals, but also due to mutational changes. For Group A Streptococci (the causative agent of scarlet fever, necrotizing fasciatis and other infections) it was shown that an insertion of 7 nucleotides in a regulator gene was sufficient to alter transcription and turn a benign Group A Streptococcus strain into a highly infectious variant [13]. 

Whole genome sequencing of sets of *S. aureus* isolates obtained from colonization and subsequent infection from the same patient may provide insight into small changes (single nucleotide polymorphisms or SNPs) in the genome that may contribute to the altered gene expression profiles during infection when compared to colonization [14]. The transfer of livestock-associated methicillin-resistant *S. aureus* (LA-MRSA) from pigs to humans is the most common change of hosts among *S. aureus* as up to 30% of the pig farmers are at one moment colonized by LA-MRSA [15]. We therefore compared the whole genome sequences of three sets of isolates from the same patients as well as a set of isolates from a colonized pig and a farmer obtained from the same farm. The data indicate that differences between the isolate from infection and the colonizing isolate exist, although one set of human isolates did not constitute a true pair. Some differences between the LA-MRSA isolates was also observed.

## Materials and Methods

### Ethical Statement

Isolates were obtained as part of routine diagnostic testing and were analyzed anonymously and the isolates, not humans, were studied. All data was collected in accordance with the European Parliament and Council decision for the epidemiological surveillance and control of communicable disease in the European community [16,17]. Ethical approval and informed consent were not required.

### Bacterial isolates

Bacterial isolates were identified as part of the FP7-HEALTH program CONCORD (Control of Community-acquired MRSA: Rationale and Development of Counteractions) [18]. The collection was not part of a structured survey with pre-defined criteria of isolate collection. It was a convenience sample composed of 568 *S. aureus* isolates obtained from infection (74%) and colonization (17%) of patients attending health-care centers and hospitals, collected within 48 hours of hospitalization. Among this set of isolates three patients were identified with pairs of isolates for which the first was an isolate from colonization and the second from an infection site. All isolates were obtained in 2005. Isolates S1800 and S1805 were obtained 11 days apart, isolates S2395 and S2396 29 days, and S2397 and S2398 17 days apart. The infection for the first set was cellulitis, whereas the latter two sets both caused a furuncle.

In addition, a set of livestock-associated methicillin-resistant (LA-MRSA) isolates were identified at the same time on a single farm. One isolate was from the nose of a colonized pig (S0462) the other from the nose of the colonized pig farmer (S0460).

### Whole sequencing and analysis

Whole genome sequencing and contig assembly for the three patients pairs were performed by BaseClear (Leiden, the Netherlands) using Illumina GA IIx technology. The LA-MRSA isolates were sequenced by KeyGene (Wageningen, The Netherlands) using 454 GS FLEX technology. The sequences have the following EMBL accession nrs: S0460: CAVH010000001-CAVH010000167; S0462: CAVX010000001-CAVX010000134; S1800: CAWB010000001-CAWB010000401; S1805: CAVY010000001-CAVY010000125; S2395: CAVU010000001-CAVU010000120; S2396: CAWA010000001-CAWA010000096; S2397: CAVV010000001-CAVV010000075; S2398: CAVW010000001-CAVW010000078.

The contigs were mapped against strain Newman (GenBank accession number AP009351)for S1800 and S1805; MSSA476 (GenBank accession number BX571857) for S2395 and S2396; and WKZ-1 (GenBank project ID 40253) for S2397 and S2398, as these strains appeared to most closely resemble the sequences of the isolates. The LA-MRSA isolates were mapped against S0385, the first sequenced LA-MRSA (GenBank accession number AM990992) [19]. Novel sequences were based on BLAST against GenBank databases. Annotations was performed using Kodon (Applied Maths, Sint-Martens-Latem, Belgium). The isolates were then compared pairwise by alignment for differences in gene content and SNPs using Kodon. Differences in gene content were confirmed by PCR.

Sequencing coverage was 9, 10.5, 96, 118, 275, 114, 306, and 178 times for S0460, S0462, S01800, S1805, S2395, S2396, S2397, and S2398, respectively. SNPs in the three pairs of human derived isolates were excluded when 1) they were present within 50 bp of the end of a contig; 2) were within 10 bp of a GGC motif [20]; 3) were present in multiple sequences >50 bp with >98% similarity; remaining potential SNPs were validated by mapping the reads. Regions smaller than 1 kb with more than 3 mutations were sequenced by conventional sequencing to determine SNP status.

SNPs in *S. aureus* isolates S0460, S4062 were identified slightly differently, because the genome was sequenced using 454 technology. SNPs were identified by mapping the 454 sequencing reads against the reference genome using GS reference mapper software (Roche) and by sequence alignment of the contigs against the reference genome using MUMmer [21]. SNPs located in polyA/T tracts were excluded. The remaining SNPs were categorized as true, possible or probable depending the following criteria: 1) supported by at least 5 reads; 2) at least 90% SNP agreement between reads; 3) SNP position in the contig is at least 50 nucleotides away from the border of the contig; 4) quality score assigned by 454 sequencing. Only if all criteria were met the SNP was deemed true, otherwise they were reported as possible or probable when one or two criteria were not met, respectively. SNPs located in mobile genetic elements not conserved among the isolates, repeat regions or paralogous genes were excluded from further analysis. We selected 20 SNPs for PCR amplification and traditional capillary sequencing in all four isolates which confirmed the criteria used to select true SNPs.

## Results

### S1800 and S1805

Both S1800 and S1805 belong to sequence type 80 (ST80) and *spa*-type t-044 indicating that both isolates belong to the European clone of CA-MRSA. Isolate S1800 was a carrier isolate whereas isolate S1805 caused an infection.

A Staphylococcal Cassette Chromosome *mec* (SCC*mec*) type IV was present in both S1800 and S1805. The region of the *mec* element also contains a Fok type II restriction methylase and restriction endonuclease, which is also present in the sequence of the first published ST80 strain [22].

Both isolates also contained a plasmid. A part of the plasmid encoding 7 proteins is present in *Streptococccus mitis* strain B6 (GenBank Acc no. FN568063.1) (Table 1). Compared to *S. mitis* B6 a number of mutations occur and some nucleotides are missing in a scattered pattern between bp 28,227-28,555 and a stretch of nucleotides lacks between bp 30,426 and 30,448 in the *S. mitis* isolate. The missing stretch is in the intergenic region between a transcriptional regulator and the aminoglycoside 3'-phosphotransferase and the scattered changes are in the streptomycin aminoglycoside 6-adenyltransferase. This may represent a novel variant of this gene. 

**Tabel 1:** Genes on the S1800 and S1805 novel *S. aureus* plasmid and present as a unit in *Streprococcus mitis* B6.

**gene product**	**% similarity at DNA level**
nucleotidyl transferase	100
methyltransferase	100
streptomycin aminoglycoside 6-adenyltransferase	93
streptothricin acetyltransferase	100
aminoglycoside 3'-phosphotransferase	99
transcriptional regulator	99
acetyl transferase	100

Comparison with the first completely sequenced ST80 (GenBank accession number CP003194) showed that it carried an additional prophage compared to S1805. This prophage was partially present in S1800. The other main difference is that the SCC*mec* of the first completely sequenced ST80 contained additional sequences (approximately 23 kb) between the *ccr* genes and *mecA*.

When S1800 and S1805 were compared for gene content major differences were observed. S1800 contained an additional prophage without any striking proteins. Both isolates contained the genes for staphylokinase and the staphylococcal complement inhibitor which are located on a prophage. The isolates also contained the genes for Panton-Valentine toxin. In addition, then gene encoding SplF protease and a stretch encoding 3 hypothetical proteins were additionally present in S1800. The S1805 contained an integrative conjugative element similar to one reported for MRSA ST398 strain S0385 [19]. In S1805 genes encoding a LPTXG-motif cell wall anchor protein, exotoxin 9, a putative lipoprotein and a hypothetical protein were present that were not found in S1805.

Initial sequence comparison between S1800 and S1805 showed the presence of 188 potential SNPs. A total of 31 potential SNPs were analyzed by conventional sequencing because they were present close to each other at seven different loci. Four of these potential SNPs could be confirmed. They were present in the ATB-binding subunit of a multidrug resistance ABC-transporter, an auto-inducer sensor protein, the sensor-histidine kinase KdpD, protein A, and the GTP-sensing transcriptional pleiotropic repressor CodY. Remarkably, three of these confirmed mutations were found in proteins involved directly or indirectly in gene regulation. However, the majority of the false-positive SNPs were present in genes with similar sequences elsewhere or within the same gene, e.g., the repeats of the protein A gene. Of the remaining 157 SNPs 69 were considered sequence artifacts, present in sequences that occurred multiple times in the genomes, or were phage encoded, leaving 88 potential SNPs. Mapping of the reads showed that 99-100% all reads for each SNP matched for these 88 potential SNPs. So, in total at least 92 true SNPs were present between S1800 and S1805. Seventeen SNPs were located at intergenic sequences, 20 were synonymous mutations and the remaining 55 SNPs were non-synonymous. These SNPs were present in a variety of proteins (Table S1). The majority of these would be involved in house-keeping, but some more interesting genes might be a type I restriction modification system protein and MutS involved in mutator phenotypes.

From these data we conclude that despite the fact that both isolates have the same ST, *spa*-type and the strongest epidemiological link that can be found (isolates from the same patient), the isolates are not clonal. 

### S2395 and S2396

The sequenced isolates (S2395 and S2396) belong to ST1 and have *spa*-type t-590. S2396 was an isolate from colonization obtained before the isolate from infection, S2395. When the isolates were compared for gene content only one major difference was noted. S2395 contained a bacteriophage that was absent in the other isolate. Both isolates contained the genes staphylokinase, staphylococcal enterotoxin A, and staphylococcal complement inhibitor which are located on a prophage. When compared to MSSA476, a completely sequenced ST1 MSSA, several significant differences were present. MSSA476 contained a SCC element encoding amongst others fusidic acid resistance [23], and two sequences both encoding two hypothetical proteins. Isolates S2395 and S2396 additionally contained a 13.5 kb sequence with partial homology to the staphylococcal pathogenicity island (SaPi) of strain RN3984 (GenBank accession number EF010993.1). Eight of the 14 genes showed more than 90% similarity with SaPibov (GenBank accession number AF217235). One gene encoded a PemK-like growth inhibitor associated with a SaPi in strain JKD6008, a ST239 member (Genbank accession nr CP002120.1). Five genes encoded hypothetical proteins without a homologue in the GenBank database. Furthermore, a second 15 kb SaPi is present. Highest similarity was found with the bovine SaPi and the SaPi of strain T0131. No remarkable genes could be identified, but three genes encoded hypothetical proteins not related to pathogenicity islands. A transposon, Tn*916*-like, encoding tetracycline was also present in both isolates but not in MSSA476. This 17.6 kb transposon was with 99% similarity also present in the first sequenced ST398 strain (S0385). The isolates and MSSA476 differed in a prophage. Finally, genes encoding an enoyl-(acyl carrier protein) reductase and two hypothetical proteins were present in the set of isolates and absent in MSSA476. 

After initial data analysis 114 potential SNPs were found between S2395 and S2396. A total of 59 potential SNPs were analyzed by conventional sequencing because they were present close to each other at 11 different loci. None of these potential SNPs could be confirmed. Of the remaining SNPs 49 were considered sequence artifacts, present in sequences that occurred multiple times in the genomes, or were phage encoded. Four of the remaining 6 potential SNPs could not be confirmed by mapping of the reads. This was most likely also due to multiple copies of the sequences since approximately half of the reads showed one nucleotide and the other half another nucleotide. The remaining 2 SNPs were confirmed by mapping the reads. One was an A to G change in an intergenic region in front of a putative peptidase in S2395 and the second was a non-synonymous G to T mutation in a gene encoding a putative membrane protein resulting in a valine to fenylalanine amino acid change.

The presence of a low number of potential SNPs and the fact that the 59 resequenced potential SNPs confirmed their absence suggest that the isolates are closely related despite a difference in prophage content. 

### S2397 and S2398

The two sequenced isolates (S2397 and S2398) belong to ST30 and have *spa*-type t-433. S2398 was an isolate from colonization obtained before the isolate from infection, S2397. Community-associated MRSA with this sequence type are considered to belong to the South-West Pacific clone. The isolates do not differ in gene content. The genomes do not contain any novel sequences. 

The differences between this pair of isolates and a representative of ST30 we sequenced before, MSSA WKZ-1 [24] were considerable. The most important differences were that WKZ-1 possessed SaPi2 encoding, amongst others, toxic shock syndrome toxin, a second SaPi structure encoding at least two putative enterotoxins, an integrative conjugative element, transposon Tn*552* encoding an ß-lactamase, an unnamed plasmid-like structure encoding amongst others arsenic resistance that is also found in MRSA252. Furthermore, two prophages differed between the pair and WKZ-1, but no virulence factors could be identified on these. Both isolates contained the prophage encoded immune evasion cluster encoding staphylokinase, staphylococcal enterotoxin A, staphylococcal complement inhibitor, chemotaxis inhibitory protein of *S. aureus*. Isolate S2397 and S2398 contained 4 additional genes on 2.8 kb DNA, but no function could be assigned to these genes. This sequence has also been observed in a number of other *S. aureus* such strains TW20 and TCH60 (GenBank accession numbers CP002120 and CP002110, respectively).

After initial data analysis 105 potential SNPs were found between S2397 and S2398. A total of 39 potential SNPs were analyzed by conventional sequencing because they were present close to each other at six different loci. None of these potential SNPs could be confirmed. Of the remaining potential SNPs 65 were considered sequence artifacts. One SNP was identified, a synonymous mutation (a to g in S2398) in a putative lipoprotein.

These data are in agreement with our hypothesis that the isolates form a pair, because only one of the potential SNPs was confirmed. In this case most likely no adaptation occurred when the strain went from carrier status to infection.

### S0460 and S0462

S0460 and S0462 belong to ST398, have *spa*-type t-011, and contain a SCC*mec* type IV. .There were no differences in gene content between the bacterial chromosomes of the two farm derived *S. aureus* ST398 isolates. Many of their characteristics are shared with the reference genome *S. aureus* ST398 isolate S0385. All isolates possessed the allelic variants of νSaα and νSaß islands described previously for ST398 and which seem to unique for this sequence type. νSaß is most notable of these, because it lacks the genes encoding type I restriction-modification system, enterotoxins, serine proteases, lantibiotic biosynthesis proteins and LukE/D hemolysins commonly found in the other *S. aureus* genomes. SaPi-S0385 encodes two excreted proteins with homology to the staphylococcal complement inhibitor (SCIN) and von Willebrand factor binding protein (Wbp) [19]. Like in isolate S0385, resistance to penicillin and tetracycline is conferred by Tn*552* and Tn*916*, encoded by *bla* and *tet*(M), respectively. A third transposon (Tn*7*-like) with unknown function(s) was present in both isolates. Furthermore, a prophage very similar to φSa6S0385 is inserted; we could not identify any known or hypothetical virulence genes on this prophage. 

Comparison with the reference strain S0385 also showed divergence between the genomes, most of which can be explained by the presence or absence of complete mobile genetic elements (MGE). The farm derived isolates all possess SCC*mec* type IV while the reference has SCC*mec* type V integrated into *orfX*. A second prophage is present in the farm isolates, but no genes with homology to known virulence factors were found. Integrated into the gene encoding for the DNA repair protein RadC is a Tn*554*-like element encoding a dihydrofolate reductase enzyme *dfr* in accordance with the trimethoprim resistance observed in the farm derived isolates. 

The two isolates were very similar and did show only 12 mutations (Table 2). Remarkably, the majority of these were non-synonymous SNP or insertions or deletions (indels) and resulted in the truncation of two genes. The genes belong to a number of different functional categories (Table 2). 

**Table 2:** Mutations between S0460 and S0462. Gene function, SNP type, nucleotide change, amino acid (AA) change and possible consequences are given.

**gene encodes**	**nucleotide change**	**AA change**
iron compound ABC transporter	t-c	S-G
intergenic	c-t	none
iron regulated protein (Isd)	t-c	Y-H
cell division protein FtsY	c-a	A-E
DNA translocase FtsK	g-a	A-T
competence/damage inducible protein CinA	t-c	L-S
hypothetical protein	g-t	X-stop*
glyoxalase/blemomycin resistance	c-t	G-D
septation ring formation regulator (EzrA)	c-t	E-K
AgrC	g-a	G-S
Protein YhgF/RNA binding S1 domain protein	g-a	Q-stop*
GTP-pyrophosphate kinase	t-c	E-G

*results in truncated gene

## Discussion

The change of the bacteria from colonizers to pathogens is accompanied by a drastic change in expression profiles [12]. A similar change probably occurs when bacteria change hosts. These changes in expression profile may be due to environmental signals, but also due to mutational changes. An example is insertion of seven nucleotides in a regulator gene of a Group A Streptococcus strain which altered transcription and turned a benign strain into a highly infectious variant [13]. The transfer of livestock-associated methicillin-resistant *S. aureus* (LA-MRSA) from pigs to humans is the most common change of hosts among *S. aureus* as up to 30% of the pig farmers are at one moment colonized by LA-MRSA [15]. We therefore investigated possible adaptations in four sets of epidemiologically strongly related isolates. Surprisingly the results were variable. Two sets of isolates (S1800/S1805 and S2395/S2396) showed differences in prophages. However, prophages can be easily lost or acquired. One can wonder whether this influenced the infectious or colonization ability of the isolates. We could not identify any described virulence factors on the prophages, but several hypothetical proteins remain that can have a function in pathogenesis. 

The more than 92 SNPs between S1800 and S1805 and a difference in prophage content make it highly unlikely that isolate S1805 originated from S1800. Therefore, the infection with S1805 was derived from a different ST80 strain. The finding of different isolates from the same patients is at odds with the common assumptions for MRSA outbreak management: isolates with the same *spa*-type and a strong epidemiological link belong to the same outbreak. Here different strains were found within the same patient. These also showed that ST80, the European CA-MRSA, is not highly clonal. Even larger differences exist when the S1800 and S1805 sequences are compared with a ST80 sequence that was published during this study [22]. This contrasts the findings for USA300. This strain has been shown to be highly clonal [25] and only recently we demonstrated some variation in gene content [26].

We conclude that the other sets are most likely pairs of isolates, because the differences for the other sets are minimal. Only one SNP in S2395/S2396 and 11 SNPs in S0460/S0462 may have an effect since they are either non-synonymous or in intergenic regions. In addition, differences with other isolates belonging to the same ST are much larger. It should be noted that a non-synonymous mutation occurred in AgrC in S0460/S0462 which is a component of the *agr* system that regulates virulence genes. Furthermore, two genes became truncated (Table 2). These changes may reflect adaptation after the change in niche. Our data are in agreement with data of Young et al [14 of 15] that 8 mutations accompanied the transition from nasal carriage to bloodstream infection in a set of isolates from one patient. Four of these mutations resulted in truncation of proteins. One of the truncations was in an *araC*-family transcriptional regulator implicated in pathogenicity. The other truncated proteins are an iron-compound binding protein/transporter, a GNAT family acetyltransferase, and a protein of unknown function. An alternative explanation for the variation found in the true pairs is pre-existing variation in the colonizing strain. The recent report of Harris et al who analyzed a MRSA outbreak showed also considerable differences in SNPs between 20 colonies from a single nasal sample [27]. It remains to be seen whether all variants are equally able to cause infections or whether particular variants are more able to cause infections and that this sequence variation enhances the ability of *S. aureus* to cause infections or colonize new hosts.

The isolates that belong to ST1 (which includes the Mid-Western USA clone), ST30 (which includes the South-West Pacific clone), and ST398 (the LA-MRSA) also show considerable differences in gene content with other members of these STs. One can wonder whether it is possible to speak about specific CA-MRSA clones with maybe the exception of USA300, which shows only little gene and sequence variation [25]. It seems more likely that SCC*mec* was introduced several times in the other STs that are linked to the well-known clones and that their pandemic spread is more limited than assumed. Further support for this comes from a study by Rolo et al [18] that the composition of the European CA-MRSA population is highly diverse. Especially the presence of a large number sporadic isolates indicates that transfer of methicillin is more frequent than expected. This is in accordance with findings of Nübel et al, who showed a rather frequent uptake of methicillin resistance by different members of the same ST [28]. Likely, most CA-MRSA clones are as diverse in their accessory genome as other *S. aureus* STs [29] and that CA-MRSA have probably arisen in the same ST at multiple occasions.

The data indicate that differences between the isolate from infection and the colonizing isolate exist. Whether these SNPs reflect true adaptations remains uncertain. It is not clear whether variation is common and whether it contributes to the ability of a strain to cause infections.

## Supporting Information

Table S1 **SNPs in strain S1800 and S1805.**
(XLSX)
